# Association of anemia with mobility capacity in older adults: a Korean nationwide population-based cross-sectional study

**DOI:** 10.1186/s12877-020-01879-z

**Published:** 2020-11-13

**Authors:** Ki Young Son, Dong Wook Shin, Ji Eun Lee, Sang Hyuck Kim, Jae Moon Yun, Belong Cho

**Affiliations:** 1 Department of Family Medicine, Asan Medical Center, 88 Olympic-ro 43 gil, Songpa-gu, Seoul, 05505 South Korea; 2 Department of Family Medicine/Supportive Care Center, Samsung Medical Center, School of Medicine, Sungkyunkwan University, Seoul, South Korea; 3 Center for Clinical Epidemiology, Samsung Advanced Institute for Health Sciences & Technology (SAIHST), Sungkyunkwan University, Seoul, South Korea; 4 Department of Family Medicine, CHA Bundang Medical Center, CHA University, Seongnam-si, Gyeonggi-do South Korea; 5 Department of Family Medicine, Bumin Hospital, Seoul, South Korea; 6 Health Promotion Center, Seoul National University Hospital, Seoul, South Korea; 7 Department of Family Medicine, Seoul National University Hospital, Seoul, South Korea; 8 Institute on Aging, Seoul National University College of Medicine, Seoul, South Korea

**Keywords:** Anemia, Timed up and go test, Older adults, Mobility capacity

## Abstract

**Background:**

Over 10% of adults aged ≥65 years have anemia, as defined by the World Health Organization (WHO). As the timed up and go (TUG) test is one of the most widely used tests of mobility, this study investigated whether anemia was associated with mobility capacity assessed using the TUG test in older adults.

**Methods:**

Subjects belonging to the Korean National Health Insurance Service-National Health Screening Cohort of the National Health Information Database were reviewed. Subjects were included if they had completed the TUG test as part of the National Screening Program for Transitional Ages in Korea. An abnormal TUG test result was defined as a time of ≥10 s and anemia was defined according to the WHO criteria as a hemoglobin (Hb) concentration of < 13.0 g/dL in men and < 12.0 g/dL in women. The association between anemia and TUG test results was evaluated using four multiple logistic regression models with different levels of adjustment. Stratified analysis according to risk factors was performed.

**Results:**

The 81,473 subjects included 41,063 (50.4%) women and 40,410 (49.6%) men. Mean TUG time was 8.44 ± 3.08 s, and abnormal TUG test results were observed in 22,138 (27.2%) subjects. Mean Hb concentration was 13.72 ± 1.41 g/dL, and 10,237 (12.6%) subjects had anemia. U-shaped associations between Hb concentration and TUG test results were observed in both sexes. Subjects with anemia were 19% more likely to have abnormal TUG test results, according to the fully adjusted model (adjusted odds ratio: 1.192, 95% confidence interval: 1.137–1.247). Similar results were observed for both sexes. Stratified analysis showed that subjects with anemia were more likely to have abnormal TUG test results regardless of risk factors.

**Conclusions:**

Individuals with anemia are more likely to have abnormal TUG test results, regardless of risk factors, than individuals without anemia. U-shaped relationships between Hb concentrations and TUG test results were observed in both sexes, although the optimal Hb concentration differed between men and women.

## Background

Anemia is a common condition in older adults and is due to multiple factors. Over 10% of adults aged ≥65 years have anemia, as defined by the World Health Organization (WHO) [1]. Anemia is caused by nutritional deficiency in one third of these individuals, by chronic illness, including renal insufficiency, in another third, and by undetermined factors in another third [1–3]. Anemia in older adults is associated with various negative health outcomes, including reduced quality of life [3–5], depressive mood and fatigue [4, 5], falls [6, 7], frailty [3, 8], impairment in ADL [4, 9–11], cognitive impairment [10], and increased mortality [12, 13].

Anemia may reduce muscle oxygenation, consequently affecting muscle strength and quality and, therefore, physical performance including mobility capacity [14, 15]. Given the importance of physical performance for health outcomes and quality of life in older adults, and the fact that anemia is both common in older adults and easily discovered in the usual primary care setting, it is important to examine the association between anemia and physical performance. Studies assessing the association between functional decline in mobility and anemia in older adults have shown that anemia is associated with impaired standing balance [4, 14, 15], slower gait speed [4, 14–16], slower chair standing [4, 14, 15], decreased muscle strength including, handgrip and knee extensor strengths [4, 15], and multidimensional loss of function [17].

The timed up and go (TUG) test is one of the most widely used tests of mobility capacity in older adults. It includes standing and walking activities commonly performed in daily life, including walking, turning, and transitions [18]. The test is easy to perform in clinical setting, and is useful to assess aspects of mobility, such as static/dynamic balance, lower extremities strength, and gait speed. Previous studies reported that TUG test predicted falls, fractures, hospital admissions due to fractures [19], disability [20], low quality of life [21], low social participation [21], complications after elective surgery in patients with cancer [22], and onset of difficulty in activities of daily living (ADL) [23].

In contrast, previous studies assessing the association between anemia and TUG test results as a mobility measure have yielded inconsistent results, with some reporting an association between anemia and impaired TUG test results (i.e., 0.14–0.36 s improvement in TUG test results with a 1 mg/dL increase in Hb; 1.5 times greater proportion of impaired TUG test results in anemic group) [16, 24], and others reporting no association [21, 25]. These studies, however, included relatively few subjects (i.e., 62, 93, 122, and 236 participants, respectively) and were limited to specific populations such as patients with hip arthroplasty or patients with anemia treated with epoetin alfa. To our knowledge, no study to date has assessed these relationships in large general populations including sufficient numbers of people with anemia.

The present study therefore evaluated the association between anemia and mobility capacity assessed using the TUG test in a large general population of adults aged 66 years who had been enrolled in the National Screening Program for Transitional Ages (NSPTA) in Korea, a nationwide representative sample of Korean individuals. We hypothesized that anemia in older adults would be associated with an increased likelihood of impaired TUG test results.

## Methods

### Study design

This study was a population-based cross-sectional study using the National Health Information Database (NHID) in Korea. The participants were 66-year-old men and women who underwent the NSPTA examination between 2007 and 2015. All assessments were performed on the day of the NSPTA examination. We aimed to compare TUG test results between normal and anemic groups using multiple logistic regression analysis.

### Data sources

The Korean National Health Insurance Service (KNHIS) is a publicly available health insurance plan that provides universal health coverage to almost all Koreans except Medicaid beneficiaries, who account for < 3% of the population. The KNHIS is responsible for the National Screening Program (NSP), a biennial health screening program. KNHIS added the NSPTA to the NSP in 2007. The purposes of addition of the NSPTA are to tailor the NSP by the age and sex of participants and to strengthen counseling after examination. Only participants aged 66 years of the NSPTA undergo TUG tests and unipedal stance tests to assess mobility capacity.

The KNHIS created the NHID. The NHID includes healthcare utilization data, the NSPTA health examination results, sociodemographic information, and mortality data of over 50 million persons in Korea [26]. The details of the NSPTA have been described elsewhere [27].

### Study population

**Fig. 1:**
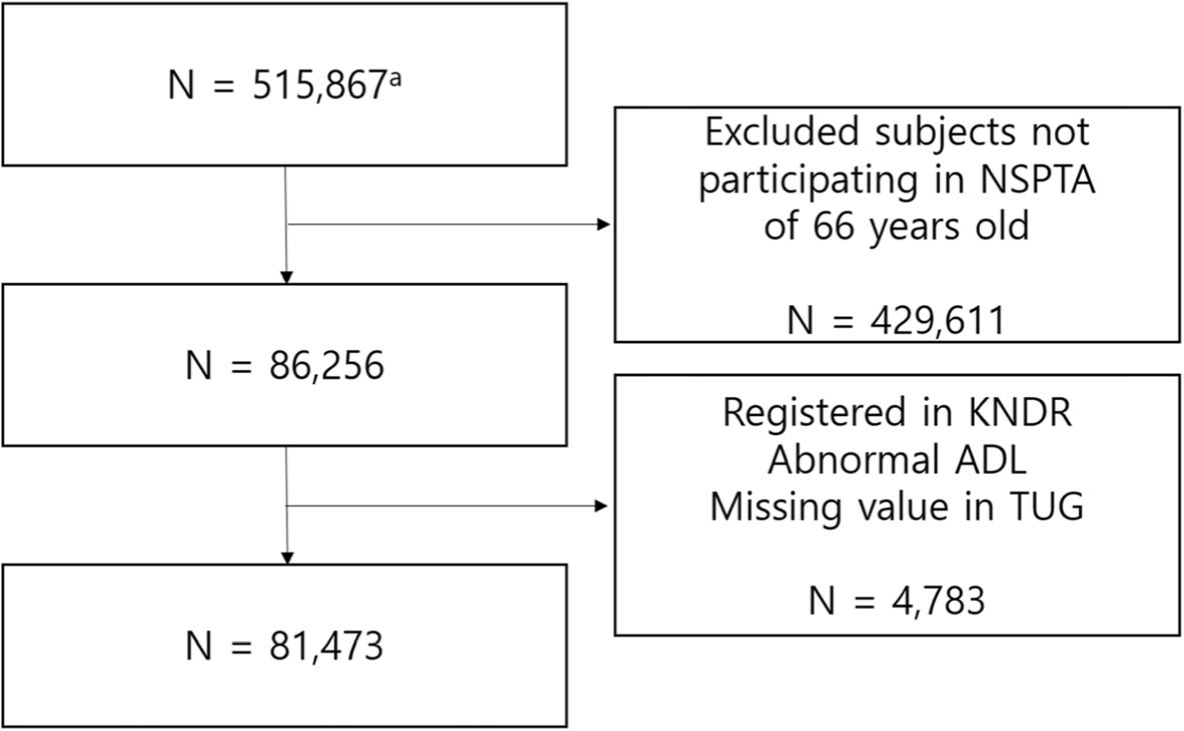
Flow of study subjects. a. Subjects of the National Health Insurance Service-National Health Screening Cohort database of the Korean National Health Insurance Service (2002–2015). *Abbreviation: NSPTA, National Screening Program for Transitional Ages in Korea; KNDR, Korean National Disability Registry; ADL, Activity of daily living; TUG, Timed up and go

The National Health Insurance Service-National Health Screening Cohort (NHIS-HEALS) database of the NHID included 515,867 randomly selected subjects, representing approximately 10% of Korean participants aged 40–79 years who participated in the NSP at least once in 2002–2003. Subjects aged 66 years who underwent the NSPTA examination between 2007 and 2015 were included. We excluded participants with disabilities at baseline (i.e., registered persons with disabilities and those with disabilities in daily activities). After excluding subjects who were registered as persons with disabilities in the Korean National Disability Registry (KNDR), those with missing TUG test results, and those with impairments in ADL, 81,473 subjects were included (Fig. 1).

The database included the participants’ demographic characteristics, previous medical history and health behaviors, and screening test results. Factors recorded included height, weight, abdominal circumference, and the results of physical function tests (i.e., TUG and unipedal stance tests) and laboratory tests.

The study protocol was approved by the Institutional Review Board of Seoul National University Hospital (IRB No. E-1703-020-836), which waived the requirement for informed consent because the KNHIS database was constructed after anonymization according to strict confidentiality guidelines. We, authors acquired permission to access the database by National Health Insurance Sharing Service (NHIS-2018-2-201). And this study used the database of NHID constructed by KNHIS, with the permission. In this reason, this study is not registered separately to clinical trial registry.

### Variables

#### Independent variables

##### Anemia

Anemia and hemoglobin (Hb) concentration were the independent variables in this study. Anemia was defined according to the WHO criteria as an Hb concentration of < 13.0 g/dL in men and < 12.0 g/dL in women [28], based on laboratory test results recorded by the NSPTA. The optimal Hb concentration associated with the fastest TUG time was determined by assessing Hb concentration as a continuous variable.

#### Outcome variable

##### Mobility capacity

The TUG test result was the outcome variable in this study, representing mobility capacity in study participants. Each subject performed the TUG test on the day of physical examination of the NSPTA, in the clinical setting of each subject’s community hospital according to the NSPTA manual. Participants were instructed to sit on a chair (i.e., a normal clinic chair without armrests), stand, walk 3 m at a comfortable speed, walk back to the chair, and sit again on the chair, all while wearing regular footwear and/or using walking aids. The time from standing to sitting again was measured once, and a time longer than 10 s was categorized as abnormal. According to the NSPTA manual, a TUG test result of > 10 s is regarded as requiring attention, and a TUG test result of > 20 s is regarded as impaired. However, a previous study using NSPTA data showed that only a small number of participants were classified as impaired when 20 s was used as the TUG test result threshold for 66-year-olds in Korea [2]. For this reason, we used 10 s as our threshold. The details of how the TUG test is conducted in the NSPTA have been described elsewhere [29].

#### Potential confounding variables

In our analysis, we included potential confounders that had been shown to affect physical performance in previous studies, and for which data were available in the database, such as chronic diseases, obesity, cognitive impairment, and depression. Data on chronic diseases, such as hypertension, diabetes mellitus, and dyslipidemia, were collected from the questionnaire and laboratory results of the NSPTA. Each condition was defined as previously described [30]. Subjects who took medications for hypertension, diabetes mellitus, or dyslipidemia were considered to have the corresponding disease. In addition, hypertension was defined as a systolic blood pressure (SBP) of ≥140 mmHg or a diastolic blood pressure (DBP) of ≥90 mmHg; diabetes mellitus as a fasting serum glucose concentration of ≥126 mg/dL; and dyslipidemia as a serum total cholesterol (TC) concentration of ≥240 mg/dL.

Body mass index (BMI), cognitive impairment, and ADL were determined as previously described [29]. BMI was calculated as weight divided by height squared (kg/m^2^). Normal BMI was defined as between 18.5 and 23 kg/m^2^ and obesity was defined as ≥25 kg/m^2^ according to Asian-specific criteria.

Cognition was assessed using the Korean Dementia Screening Questionnaire-Cognition (KDSQ-C). The KDSQ-C is a self-administered, consisting of 15 items, each rated on a three-point Likert scale (0, 1, or 2, with a higher score considered worse). When participants’ composite score ≥ 6, they were considered as cognitively impaired. The KDSQ-C is validated questionnaire [31] and integrated in the NSPTA questionnaire.

The NSPTA questionnaire included six ADL items, which derived from the Korean versions of the ADL (K-ADL) and Instrumental ADL (K-IADL) questionnaires [32]. Following four items derived from the K-ADL: “Do you bathe by yourself without help?,” “Do you dress by yourself without help?,” “Do you eat by yourself without help if a meal is prepared?,” and “Do you go to the toilet by yourself without help?” Following two items derived from the K-IADL: “Do you prepare your own meals by yourself without help?” and “Do you go outside by yourself to places within walking distance?” If participants answered “No” to one or more of these questions, they were classified as having impairments in ADL.

Three questions, which derived from the validated Korean version of the Geriatric Depression Scale, were used to assess depressive mood [33]. The questions were: “Do you feel that your activity or desire has decreased recently?,” “Do you feel that you are currently useless?,” and “Do you feel that you are currently hopeless?” If participants answered “Yes” to any of these questions, they were categorized as depressed.

### Statistical analysis

Baseline characteristics are expressed as frequencies and percentages. Continuous variables are reported as mean ± standard deviation (SD), and categorical variables as frequencies and percentages.

Multiple logistic regression models were used to evaluate the association between TUG test results and anemia. In these analyses, all variables were expressed as categorical variables as defined above. Four models were built for these analyses: a crude model and three adjusted models. Model 1 was adjusted for sex; Model 2 was adjusted for sex, depressive mood, and cognitive impairment; and Model 3 was adjusted for all the factors in Model 2, as well as BMI, chronic diseases (i.e., hypertension, diabetes mellitus, dyslipidemia), and creatinine concentration. Odds ratios (OR) and 95% confidence intervals (CI) were calculated for each model.

Participants with abnormal TUG test results thought to indicate risk were identified by stratified analyses in Model 3, with participants stratified by sex, depressive mood, cognitive impairment, obesity, chronic diseases, and cardiovascular diseases.

In addition, we fitted fractional polynomial prediction plots with 95% CI to demonstrate the prediction of TUG test results according to Hb concentrations, expressed as continuous variables.

Statistical analyses were performed using Stata software (version 15.1; StataCorp, College Station, Texas). A *P*-value of < 0.05 was considered significant.

## Results

### Baseline characteristics of participants

The NHIS-HEALS database included 515,867 subjects. Of the potentially eligible participants, we excluded 429,611 participants who did not participate in the NSPTA for 66-year-olds. Of the remaining 86,256 participants, we excluded those with disabilities at baseline, those who were registered in the KNDR (*n* = 1255), and those with impairments in ADL (*n* = 3448). In addition, of the remaining 81,531 participants, we excluded those who had missing TUG test results (*n* = 58). Ultimately, 81,473 subjects were included (Fig. 1).

These 81,473 subjects included 41,063 (50.4%) women and 40,410 (49.6%) men. Mean TUG time was 8.44 ± 3.08 s, with 22,138 (27.2%) subjects having abnormal TUG test results (≥10 s). All participants in this study were aged 66 years, because only 66-year-olds underwent the TUG test in the NSPTA. Mean Hb concentration was 13.72 ± 1.41 g/dL, with 10,237 (12.6%) participants having anemia. Mean BMI was 24.2 ± 3.0 kg/m^2^, with 1678 (2.1%) subjects being underweight (BMI < 18.5 kg/m^2^) and 32,143 (37.0%) being obese (BMI ≥ 25.0 kg/m^2^).

Approximately 20% of participants reported having depressive mood and 15% were cognitively impaired. Approximately 70% had hypertension, while approximately 26 and 28% had diabetes and dyslipidemia, respectively. Only 0.8% had cardiovascular disease (Table 1).

**Table 1:** Baseline characteristics of study subjects

	Total	Timed up and go results	
		Normal	Abnormal
	N (%)	N (%)	N (%)
Sex (female)	41,063 (50.4)	28,941 (48.8)	12,122 (54.8)
Timed up and go test result (s)	8.44 ± 3.08	7.20 ± 1.49	11.76 ± 3.73
Hemoglobin (g/dL)*	13.72 ± 1.41	13.77 ± 1.40	13.58 ± 1.43
Anemia	10,237 (12.6)	7086 (11.9)	3151 (14.2)
BMI (kg/m^2^)**			
Underweight (< 18.5)	1678 (2.1)	1211 (2.0)	467 (2.1)
Normal weight (18.5–25)	49,652 (60.9)	36,546 (61.6)	13,106 (59.2)
Overweight (≥ 25)	32,143 (37.0)	21,578 (36.4)	8565 (38.7)
Depressive mood	16,458 (20.3)	11,342 (19.2)	5116 (23.2)
Cognitive impairment	12,247 (15.1)	8488 (14.4)	3759 (17.1)
Hypertension	58,695 (71.7)	42,023 (70.8)	16,372 (74.0)
Diabetes mellitus	21,065 (25.9)	14,638 (24.7)	6427 (29.0)
Dyslipidemia	23,026 (28.3)	16,149 (27.2)	6877 (31.1)
Cardiovascular disease	649 (0.8)	484 (0.8)	165 (0.8)

Results are reported as mean ± standard deviation or as number (%).

*Anemia was defined as a hemoglobin concentration of < 13 g/dL in men and < 12 g/dL in women

**Abbreviation: *BMI* Body mass index

### Association between anemia and timed up and go test results

**Fig. 2:**
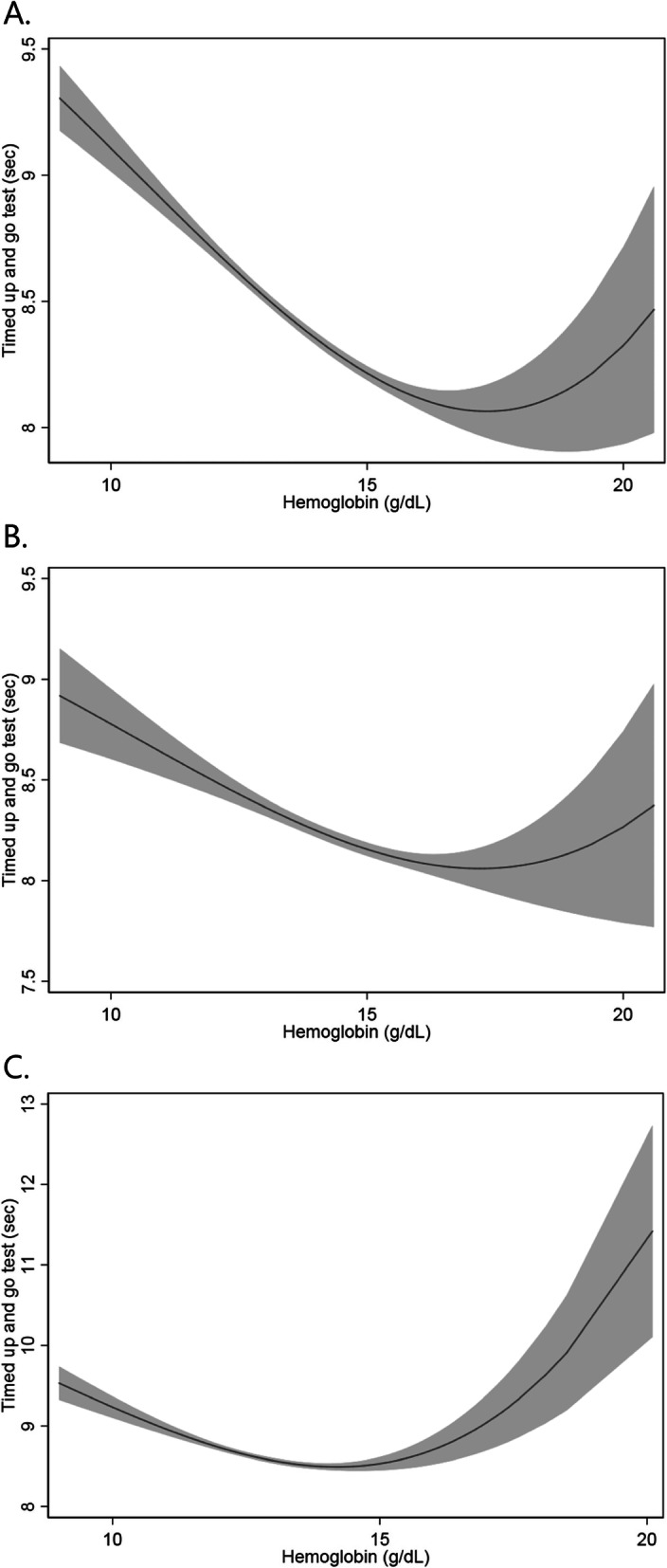
Fractional polynomial prediction plots with 95% confidence interval of association of timed up and go test results with hemoglobin concentration in **a**) all participants, **b**) men, and **c**) women. Gray areas depict 95% confidence intervals

U-shaped associations between Hb concentration and TUG test results were observed in both sexes. Optimal TUG test results were obtained at approximately 16.0–17.0 g/dL Hb in men, and at approximately 13.0–15.0 g/dL in women (Fig. 2).

Of the 71,236 subjects with non-anemic Hb concentrations, 18,987 (26.7%) had abnormal TUG test results, compared with 3151 (30.8%) of the 10,237 subjects with anemia.

People with anemia were 22% more likely to have abnormal TUG test results than people without anemia, according to the crude model (OR: 1.224, 95% CI: 1.170–1.280), and were 19% more likely to have abnormal TUG test results according to the fully adjusted model (adjusted OR (aOR): 1.192, 95% CI: 1.137–1.247).

Similar results were found in both sexes. Anemic men were 16% more likely (aOR: 1.160, 95% CI: 1.078–1.249) and anemic women were 21% more likely (aOR: 1.210, 95% CI: 1.141–1.284) to have abnormal TUG test results, according to the fully adjusted model (Table 2).

**Table 2:** Associations between anemia and timed up and go test results

	Anemia	Total (N)	Abnormal TUG** Result (N)	Crude	Model 1*	Model 2*	Model 3*
				OR** (95% CI)	aOR** (95% CI**)	aOR (95% CI)	aOR (95% CI)
Total	All	81,473	22,138				
	Normal	71,236	18,987	Ref	Ref	Ref	Ref
	Anemic	10,237	3151	1.224 (1.170–1.280)	1.195 (1.142–1.250)	1.188 (1.135–1.243)	1.192 (1.137–1.247)
Sex							
Male	All	40,410	10,016				
	Normal	36,290	8876	Ref	N/A	Ref	Ref
	Anemic	4120	1140	1.182 (1.099–1.270)		1.169 (1.087–1.257)	1.160 (1.078–1.249)
Female	All	41,063	12,122				
	Normal	34,946	10,111	Ref	N/A	Ref	Ref
	Anemic	6117	2011	1.203 (1.135–1.275)		1.200 (1.132–1.272)	1.210 (1.141–1.284)

*Model 1: Crude model + adjustment for sex

Model 2: Model 1 + adjustment for depressive mood and cognitive impairment.

Model 3: Model 2 + adjustment for BMI, hypertension, diabetes mellitus, dyslipidemia, and creatinine level.

**Abbreviations: *TUG* Timed up and go test, *OR* Odds ratio, *aOR* Adjusted odds ratio, *CI* Confidence interval, *N/A* Not applicable

#### Stratified analysis

**Fig. 3:**
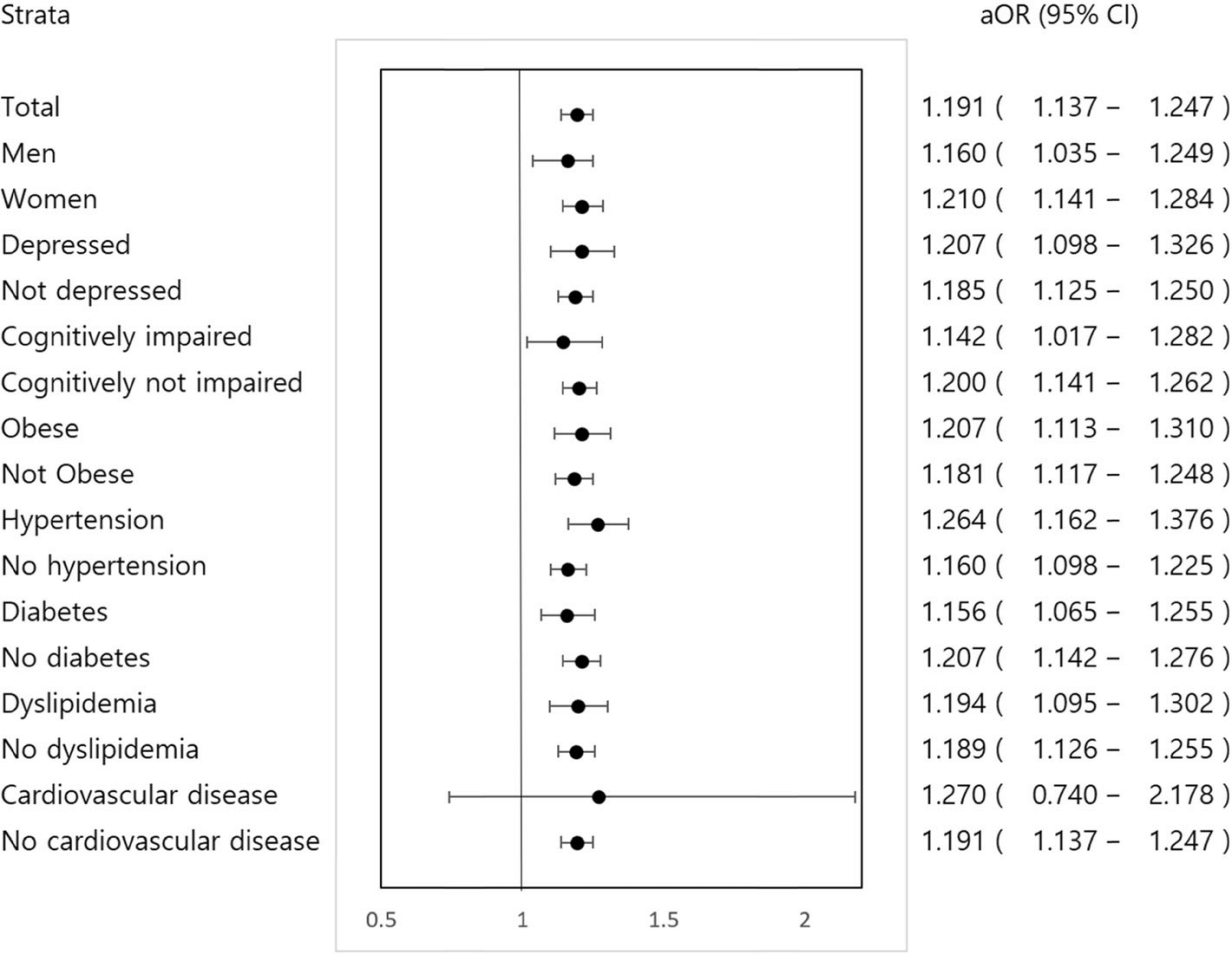
Forest plots showing stratified analyses of the association of timed up and go test results with anemia. *Abbreviation: aOR, adjusted odds ratio; CI: confidence interval; TUG, timed up and go

Stratified analysis showed that participants with anemia were more likely to have abnormal TUG test results regardless of risk factors (e.g., sex, depressive mood, cognitive impairment, obesity, and chronic diseases), except for participants with cardiovascular disease (Fig. 3).

## Discussion

This large general-population-based study evaluated the association between anemia and TUG test results in a nationally representative sample of 66-year-old subjects in an Asian country. The use of a fully adjusted model showed that anemia was associated with abnormal TUG test results and that Hb concentrations and TUG test results had a U-shaped relationship, with the Hb concentration associated with optimal TUG test results differing between men and women. Stratified analysis found that the association between anemia and TUG was independent of risk factors, including sex, depressive mood, impaired cognition, obesity, and chronic diseases.

A previous study of 100 inpatients aged ≥70 years reported that anemia and low Hb level were associated with multidimensional loss of function, in particular a 2.32-times higher likelihood of abnormal TUG test results [23]. However, studies assessing the association between anemia and TUG test results have yielded inconsistent results. For example, a community-based study in Iceland found that a 1 mg/dL increase in Hb concentration was associated with a 0.14-to-0.36 s improvement in TUG test results and predicted response to 12-week resistance exercise [22]. Similarly, a study of 93 community-dwelling older adults in Egypt found that anemia was associated with increased rates of abnormal TUG test results (64.3% of participants with anemia vs. 32.9% of participants without anemia) [24]. However, treatment of chronic anemia with epoetin alfa increased Hb concentration by 2 g/dL and improved fatigue and subject quality of life, but did not improve TUG test results [11]. Similarly, a study of patients who underwent hip arthroplasty reported that differences in Hb concentration from before to after surgery were not associated with TUG test results [25]. These previous studies, however, included relatively few subjects and were limited to specific populations, whereas the present study used a large, nationally representative sample of Asian adults aged 66 years. Therefore, this study provides evidence to overcome the limited generalizability of the relationship between anemia and TUG test results, especially in general populations of young-old individuals. However, because the present study was cross-sectional in design, its results do not indicate a causal relationship between anemia and TUG. Hence, large longitudinal studies are needed to evaluate this association.

In the present study, Hb concentrations and TUG test results were found to have a U-shaped relationship, with the best TUG test results obtained at approximately 16.0–17.0 g/dL Hb in men, and at approximately 13.0–15.0 g/dL in women. Because normal Hb concentration ranges are 14–18 g/dL in men and 12–16 g/dL in women according to the WHO criteria [28], these results indicate that the optimal range of Hb concentration for mobility capacity, determined using the TUG test results, is higher than the lower limit of normal Hb concentrations. This finding is consistent with a previous study showing that anemia is associated with mobility difficulty in community-dwelling women [34]. A U-shaped relationship was also observed between Hb concentration and mobility in the aforementioned study, with mobility difficulty being significantly lower at an Hb concentration of 13.5 g/dL than at 12.0 g/dL [34].

Owing to the cross-sectional design of this study, it was not possible to evaluate whether the association between anemia and TUG test results was causative. Anemia may not be an independent risk factor, but rather a marker for other risk factors for reduced physical performance, such as comorbid chronic diseases. However, a stratified analysis found that the association between anemia and TUG test results was robust regardless of risk factors, except for cardiovascular and cerebrovascular diseases, suggesting that anemia may be independently associated with TUG test results. This finding suggests that caution should be exercised regarding possible physical performance deterioration when a physician identifies an older adult with anemia or low-normal Hb concentration, which is a commonly identified condition in a primary care setting.

The exact mechanism by which anemia negatively affects physical performance remains incompletely understood. Anemia may reduce muscle oxygenation, consequently affecting muscle strength and quality and, therefore, physical performance. This hypothesis has been applied to the association between other vascular conditions causing muscular hypoperfusion (i.e., diabetes mellitus, peripheral vascular disease, and cardiovascular disease) and reduced physical performance in older adults. Alternatively, increased chronic inflammation may result in a greater decline in physical performance [35]. This hypothesis is supported by the identification of increased C-reactive protein concentrations in older adults, observed in another study population [36]. Additional studies are required to determine the mechanism linking anemia and physical performance. Furthermore, there appears to be a general optimal range of Hb concentration for physical performance, which means that no proportional enhancement of performance is observed above a certain concentration of Hb. This is supported by the findings of a study involving elite athletes, in which Hb concentration was not titrated toward the upper allowed limit for performance enhancement [37]. This may explain the U-shaped relationship between Hb concentration and TUG test results.

Much like studies assessing the association between anemia and TUG test results, studies assessing the association between anemia or low Hb level and falls have yielded inconsistent results. A prospective general US population-based study reported that low Hb concentration was a risk factor for recurrent falls [13], whereas two German studies reported no association between anemia and risk of falling [12, 38]. Because falls are an important health outcome in older adults and the TUG test is regarded as useful in assessing fall risk, larger longitudinal studies are required to confirm the association between anemia and falls, including the role of the TUG test in this association.

Because participants in this study participated in the NSP voluntarily, there is a possibility of selection bias, in that participants were healthy enough to travel to the NSP facility and concerned enough about their health to do so, perhaps more so on both counts than non-participants. We cannot examine the differences between participants and non-participants in the NSP because there is a lack of data on non-participants in the program. However, it is not probable that this bias affects potential participants differently according to their anemia status, because anemia is not among the factors affecting participation in the NSP in this relatively healthy population after excluding people with disabilities. Furthermore, although participants with TUG test results had increased rates of diabetes mellitus, dyslipidemia, and depressive mood (data not shown), compared with participants of the same age who did not have TUG test results, there were no evident differences in Hb concentration or rate of anemia between these two groups (13.7 g/dL vs. 13.7 g/dL, and 12.5% vs. 11.2%, respectively).

This study had several limitations, including its cross-sectional design, which prevented us from answering some of the questions that we have discussed, based on the results of this study. For this reason, we have only speculated about the answers to these questions, based on the findings of this study and previous studies. Longitudinal studies are needed to evaluate this association. In addition, the study population included only 66-year-old Korean men and women, preventing assessment of the association between TUG test results and outcomes in different age and ethnic groups. However, this large general-population study on the association between anemia and TUG test results overcame the limitation of generalizability present in previous studies involving small numbers of participants and specific populations. In addition, because the TUG test was performed in a clinical setting according to the NSPTA manual, there were procedural variations in the TUG test measurements, such as the type of chair used. Moreover, cut-off value (10 s) for TUG in NSPTA manual is not strictly based on scientific data, but on expert consensus in Korea. There are many different possible cut-off values for TUG (e.g. 12 s, 15 s), and the effect size or association could be altered if we used different cut-off value. When we applied 12 s and 15 s as cut-off in Model 3, we found aORs were 1.268 (95% CI: 1.192–1.285) and 1.204 (95% CI: 1.110–1.308), respectively. (Data not shown) The limitation of the database prevented adjustment for risk factors such as education and dietary intake. In addition, although there is no single consensus on the threshold value for the TUG test, we used 10 s as the threshold of impairment because participants were young-old and only a small number (730/81,473) of participants were classified as impaired when 20 s was used as the threshold. It may be arguable whether 10 s is the best threshold for this population. Lastly, because the NHID is fundamentally a database for medical claims, some diagnoses may have been misclassified. However, every claim in this dataset was audited by the Korean Health Insurance Review and Assessment before payment, making the misclassification of diagnoses improbable.

## Conclusions

Individuals with anemia are more likely to have abnormal TUG test results than individuals without anemia, regardless of risk factors. Hb concentrations and TUG test results have a U-shaped relationship, although optimal Hb concentrations differ between men and women.

## Data Availability

The dataset generated and analyzed during this study is available from the National Health Insurance Sharing Service. However, the authors have no right to share or provide these data. Information on how to request these data is available at https://nhiss.nhis.or.kr/bd/ab/bdaba021eng.do. The details and costs of the database are described at https://nhiss.nhis.or.kr/bd/ab/bdaba022eng.do. To request the database, available only in Korean, visit https://nhiss.nhis.or.kr/bd/ay/bdaya001iv.do. The questionnaire used in this study is not available in English. A Korean version of the questionnaire can be downloaded from. http://www.law.go.kr/admRulLsInfoP.do?chrClsCd=&admRulSeq=2200000012541#AJAX.

## Abbreviations

ADL: Activities of daily living
aOR: Adjusted odds ratio
BMI: Body mass index
CI: Confidence interval
DBP: Diastolic blood pressure
Hb: Hemoglobin
IADL: Instrumental activities of daily living
KDSQ-C: Korean Dementia Screening Questionnaire-Cognition
KNDR: Korean National Disability Registry
KNHIS: Korean National Health Insurance Service
NHID: National Health Information Database
NHIS-HEALS: National Health Insurance Service-National Health Screening Cohort
NSP: National Screening Program
NSPTA: National Screening Program for Transitional Ages
OR: Odds ratio
SBP: Systolic blood pressure
SD: Standard deviation
TC: Total cholesterol
TUG: Timed Up and Go
WHO: World Health Organization
